# Medicinal Mushrooms: Their Bioactive Components, Nutritional Value and Application in Functional Food Production—A Review

**DOI:** 10.3390/molecules28145393

**Published:** 2023-07-14

**Authors:** Paulina Łysakowska, Aldona Sobota, Anna Wirkijowska

**Affiliations:** Department of Plant Food Technology and Gastronomy, University of Life Sciences in Lublin, Skromna 8 Street, 20-704 Lublin, Poland; paulina.lysakowska@up.lublin.pl (P.Ł.); anna.wirkijowska@up.lublin.pl (A.W.)

**Keywords:** medicinal mushrooms, bioactive compounds, polysaccharides, functional foods, nutraceuticals, new generation foods, (1,3)(1,6)-β-d-glucans, polyphenols, antioxidant activity

## Abstract

Medicinal mushrooms, e.g., Lion’s Mane (*Hericium erinaceus* (Bull.) Pers.), Reishi (*Ganoderma lucidum* (Curtis) P. Karst.), Chaga (*Inonotus obliquus* (Ach. ex Pers.) Pilát), Cordyceps (*Ophiocordyceps sinensis* (Berk.) G.H. Sung, J.M. Sung, Hywel-Jones and Spatafora), Shiitake (*Lentinula edodes* (Berk.) Pegler), and Turkey Tail (*Trametes versicolor* (L.) Lloyd), are considered new-generation foods and are of growing interest to consumers. They are characterised by a high content of biologically active compounds, including (1,3)(1,6)-β-d-glucans, which are classified as dietary fibre, triterpenes, phenolic compounds, and sterols. Thanks to their low-fat content, they are a low-calorie product and are classified as a functional food. They have a beneficial effect on the organism through the improvement of its overall health and nutritional level. The biologically active constituents contained in medicinal mushrooms exhibit anticancer, antioxidant, antidiabetic, and immunomodulatory effects. In addition, these mushrooms accelerate metabolism, help fight obesity, and slow down the ageing processes thanks to their high antioxidant activity. The vast therapeutic properties of mushrooms are still not fully understood. Detailed mechanisms of the effects of medicinal mushrooms on the human organism still require long-term clinical studies to confirm their nutraceutical effects, their safety of use, and their dosage. Medicinal mushrooms have great potential to be used in the design of innovative functional foods. There is a need for further research on the possibility of incorporating mushrooms into food products to assess the interactions of their bioactive substances with ingredients in the food matrix. This review focuses on the properties of selected medicinal mushrooms and their effects on the human organism and presents current knowledge on the possibilities of their use in the production of functional foods.

## 1. Introduction

For many years, mushrooms have accompanied humans both as food and medicine. Data from the literature indicate that, with the onset of hunting, mushrooms began to play an important role in the human diet [1]. Fruiting bodies, i.n., the visible part above the substrate commonly referred to as the mushroom, are the edible elements of some filamentous fungi [2]. Fungi form a separate kingdom alongside the kingdoms of prokaryotes, eukaryotes, plants, and animals [3]. About 2.2–3.8 million species of fungi in the world have been identified, of which 150,000 species have been described, 2000 species are considered edible, and over 200 species of wild mushrooms are considered medicinal [4,5]. Edible mushrooms, unlike medicinal mushrooms, are mainly consumed as fresh mushrooms with fruiting bodies or dried products. They can also be consumed as boiled, fried, roasted, soups, tinctures, teas, and many different dishes, while medicinal mushrooms are mostly used in biopharmaceutical applications in powdered, loose, or liquid extract forms [6]. In culinary terms, mushrooms are wrongly classified as vegetables and are informally categorised as ‘white vegetables’ [7]. According to the USDA (United States Department of Agriculture), they can be used as a substitute for vegetables in the diet at a ratio of 1:1 (USDA, 2022). Due to their content of biologically active compounds with beneficial health effects, medicinal mushrooms have been used worldwide in folk medicine for centuries. They are particularly popular in Asian countries, e.g., China, Japan, Taiwan, and Korea. Due to the presence of numerous biologically active compounds, including polysaccharides, proteins, peptides, terpenoids, polyphenols, vitamins, and mineral elements, they are ascribed, e.g., anti-cancer, anti-inflammatory, antioxidant, hypocholesterolemic, hypoglycaemic, and immunomodulatory effects [8,9] However, it should be remembered that the consumption of medicinal mushrooms is not always advisable. The safety of their use during pregnancy, lactation, and in children is still poorly reported. The selected bioactive compounds found in mushrooms may potentially limit the absorption of nutrients, trace elements, and vitamins. As a result, it is recommended that the elderly and children avoid the excessive consumption of mushrooms. Additionally, individuals taking medications or herbs should exercise caution when using mushrooms due to the potential for interactions with their bioactive compounds.

The chemical profile of medicinal mushrooms varies according to species, strain, cultivation conditions (cultured or growing wild) [10], the degree of maturity [11], and the proportion of individual anatomical parts in the total mass of the mushroom [12]. This is largely determined by environmental (access to water, light, UV radiation) [9,13] and biological (type of substrate/host, presence of competing fungi) factors. Song et al. [8] compared the chemical composition and functional properties of wood-cultured and sack-cultured Shiitake (*Lentinula edodes* (Berk.) Pegler) and proved that the wood-cultured fungus had a higher content of terpenoids and phenolic components and concurrently exhibited higher antioxidant and hypoglycaemic potential compared to the sack-cultured Shiitake (*Lentinula edodes* (Berk.) Pegler). In the case of Chaga (*Inonotus obliquus* (Ach. ex Pers.) Pilát), which is a parasite of various deciduous trees, only sclerotia derived from birch trunks have contained tree-specific compounds (betulin and betulinic acid) showing anticarcinogenic activity. Equally great importance for the chemical composition and health-promoting potential of medicinal mushrooms is ascribed to the world region from which they originate [14,15]. Chaga (*Inonotus obliquus* (Ach. ex Pers.) Pilát) sclerotia collected in France, Ukraine, and Canada were characterised by their different contents of betulin, betulinic acid, and inotodiol and showed differential biological activity in different cancer cells [15]. The bioactive substances present in fungi are primary and secondary metabolites that can be synthesised in response to specific environmental stimuli [9,13]. Their content depends on the species of fungus and their growing conditions [10,16]. However, Peng and Shahidi [17] emphasise that the cultivation of medicinal mushrooms in standard conditions offers the possibility to stimulate the synthesis of selected biologically active substances and yields raw materials with a reproducible chemical composition, comparable biological effects, and greater health safety (with a lower content of heavy metals, which are often found in excess in wild mushrooms growing in polluted environments).

The existence of a huge number of medicinal mushroom species with their diverse chemical composition and content of biologically active compounds and thus multidirectional effects on the human organism could make mushrooms objects of growing consumer interest. In 2020, the size of the global mushroom market was 14.35 million tonnes; it is estimated to grow to 24.05 million tonnes in 2028. The most popular mushrooms among consumers include Reishi (*Ganoderma lucidum*), Lion’s Mane (*Hericium erinaceus*), Chaga (*Inonotus obliquus* (Ach. ex Pers.) Pilát), Turkey Tail (*Trametes versicolor* (L.) Lloyd)), Shiitake (*Lentinula edodes* (Berk.) Pegler), and Cordyceps (*Ophiocordyceps sinensis* (Berk.) G.H. Sung, J.M. Sung, Hywel-Jones and Spataforaprior name *Cordyceps sinensis*). It is, therefore, expedient to compile and systematise existing knowledge on the most popular medicinal mushrooms, compare their functional potential, and discuss the possibilities of their use in the food industry.

## 2. Nutritional Value and Bioactive Components

Due to their high-water content (about 80–90%), the fruiting bodies of medicinal mushrooms are low in calories (50–70 kcal/100 g) [18]. After drying, their moisture content is at the level of about 3–13% [12,19,20]. The chemical composition of medicinal mushrooms is shown in Table 1. These mushrooms are a source of such nutrients as carbohydrates (65.6–87.13% d.b.), dietary fibre (16–53% d.b.), protein (3.87–37.4% d.b.), minerals (6.2–9.7% d.b.), and fats (1–5.62% d.b.) [21].

### 2.1. Polysaccharides

Carbohydrates present in fungi are represented by monosaccharides (glucose, fructose, galactose), alcohol sugars (mannitol), oligosaccharides (trehalose, malezitose), and polysaccharides, among which homopolysaccharides (glucans, chitin, glycogen) and heteropolysaccharides (xylomannan, α-(1→4)-d-glucopyranosyl and β-(1→6)-d-galactopyranosyl with branches at *O*-6 of glucose and *O*-2 of galactose, 6-*O*-galactopyranoses substituted at *O*-2 by 3-*O*-d-mannopyranosyl-L-fucopyranosyl, α-d-mannopyranosyl, and α-L-fucopyranosyl, α-(1→3)-linked galactose, with β-(1→4),(1→6)-glucose and fucose branches, mucilage composed of glucose and galactose can be distinguished). Carbohydrates can also occur in complexes with other compounds (e.g., proteins) and may include various sugar subunits [29,30,31]. Depending on their structure, bond type, and molecular weight, carbohydrates have different functional properties. The main indigestible polysaccharides present in fungi are chitin and β-d-glucans. They are composed of sugar units that are linked by β-glycosidic bonds. The monomer in chitin is β-glucosamine and is linked by 1-4-β-glycosidic bonds, while β-glucans are made up of glucopyranose molecules. The molecules linked by β-(1,3) and β-(1,4) glycosidic bonds form linear segments to which side chains are attached via β-(1,6) glycosidic bonds [29]. These compounds are classified as dietary fibres. They are found in fungal fruiting bodies and in fungal cells at both the vegetative and generative stages of ontogenesis and play a structural role in co-forming cell walls. A special physiological role is attributed to β-d-glucans and complexes of these compounds with proteins [32,33].

Their structure takes the form of a single helix, a triple helix, or a random helix. Depending on their molecular weight, the type of β-glycosidic bonds present in the molecule, and the chain conformation, these compounds exhibit different functional properties [34]. Beta-glucans with a triple helix structure show a greater ability to inhibit tumour growth than β-glucans in a single helix form [35]. As reported by Sletmoen and Stokke [36] and Brown and Gordon [37], compounds with a higher molecular weight and lower water solubility are more potent immunostimulators, while β-glucans with a low MW and a short side chain are considered less active. On the contrary, Rop et al. [34] found that water-soluble β-glucans had stronger immunomodulatory properties than water-insoluble β-glucans. Macrophages mainly act as antigen-presenting immune cells, which contribute to the immunomodulatory effect of β-glucans by stimulating the fight against bacteria and viruses. High molecular weight molecules stimulate the action of specific lymphocyte—NK cells, which show cytotoxic effects against tumour cells.

In addition, they upregulate the expression of cytokines that are associated with immune response, including interferon-γ, TNF-α, IFN-g, IL-1, and IL-12, which inhibit tumour cell proliferation and induce their apoptosis, thereby exerting anti-tumour, antibacterial, and antiviral effects [23,37,38]. These compounds are often used as adjuvants in traditional cancer chemotherapy [39,40,41,42,43,44].

The content of β-glucans in mushrooms varies between 3.79% d.b. for Cordyceps (*Ophiocordyceps sinensis* (Berk.) G.H. Sung, J.M. Sung, Hywel-Jones and Spatafora) and 60.79% d.b. for Turkey Tail (*Trametes versicolor* (L.) Lloyd) (Table 2) [44,45]. In general, edodes are a better source of these compounds than caps. The Shiitake (*Lentinula edodes* (Berk.) Pegler) mushroom is a rich source of β-glucans [34]. It takes its specific name from its β-glucan lentinan, which stimulates immune cells to attack cancer cells. Lentinan enhances the production of T lymphocytes and can potentiate the effect of AZT (3′-Azido-3′-deoxythymidine) in the anti-viral treatment of AIDS [27]. Its positive effects have been proved in the treatment of, e.g., glioma (human astrocytoma U251 cells) [46], breast cancer [47] and liver cancer [48]. In turn, the Turkey Tail (*Trametes versicolor* (L.) Lloyd) contains characteristic proteoglucans. One of these is crestin, also known as polysaccharide-K (PSK), which contains about 25–38% of the protein in the molecule. This proteoglucan is effective in the treatment of, e.g., gastric, oesophageal, colon, rectal, and lung cancer [29].

Another type of glucan that is complexed with protein present in Turkey Tail (*Trametes versicolor* (L.) Lloyd) mushrooms is called PSP (Poly Saccharo Peptide) and activates immune cells by increasing the production of cytokines, chemokines, histamine, and prostagladin E. It reduces the detrimental effects of chemotherapy by alleviating fatigue, loss of appetite, vomiting, a dry mouth, and other related discomforts [49]. In addition to β-glucans, biological activity has also been attributed to poly- and monosaccharides occurring in complexes with other compounds. An example is the cordycepin present in cordyceps (*Ophiocordyceps sinensis* (Berk.) G.H. Sung, J.M. Sung, Hywel-Jones, and Spatafora). Its chemical structure resembles that of the nucleoside adenosine (ribose + adenine sugar); however, it lacks one hydroxyl group at position three of the five-membered ring of the ribose moiety. Adenosine is involved in DNA and/or RNA synthesis in cells. Thanks to its analogy to adenosine, cordycepin can build into the RNA and DNA structures of bacteria and viruses and interfere with the biosynthesis and modification of nucleic acids, thereby limiting the growth of these microorganisms. It increases the proliferation and secretion of T and B lymphocytes and has anti-inflammatory effects through a reduction in the expression of pro-inflammatory cytokines and chemokines. Additionally, it inhibits platelet aggregation and shows suppressive properties against tumour cells [50]. The positive effects of polysaccharides as well as other phytochemicals present in mushrooms, are shown in Table 3.

### 2.2. Proteins

In addition to polysaccharides, proteins, and peptides are important bioactive components that are present in mushrooms. Their content ranges widely from 4.6 to 56.3 g/100 g and is mainly determined by the mushroom species. Of the mushrooms discussed, Lion’s Mane (*Hericium erinaceus* (Bull.) Pers.), Cordyceps (*Ophiocordyceps sinensis* (Berk.) G.H. Sung, J.M. Sung, Hywel-Jones and Spatafora), and Shiitake (*Lentinula edodes* (Berk.) Pegler) have the highest protein content (more than 20%) [23]. The amino acid composition and sequence and the length of the polypeptide chain can determine the specific biological activity of these compounds. They are most commonly ascribed to hypotensive, angiotensin-converting enzyme (ACE) inhibition, antioxidant, anticancer, antiviral, and antibacterial activities [63]. The most important bioactive fungal proteins include lectins (glycoproteins), immunomodulatory proteins, and proteins with enzymatic activity, e.g., nucleases, ribonucleases, laccase, and ergotionein [64]. Lectins increase insulin secretion and contribute to lowering blood sugar levels. In addition, they activate the immune system and show chemo-preventive effects against various types of cancer, e.g., hepatocellular carcinoma [65,66]. These compounds are present, e.g., in Reishi (*Ganoderma lucidum*). This type of protein, named TVC, was also isolated by Li et al. [67] from the fungus *Trametes versicolor*. As demonstrated by the authors, TVC increases the proliferation of human peripheral blood lymphocytes and is responsible for the increased necrosis of alpha tumour cells that are induced by mouse macrophages [67]. A characteristic low molecular weight peptide (cordymin) is present in cordyceps (*Ophiocordyceps sinensis* (Berk.) G.H. Sung, J.M. Sung, Hywel-Jones and Spatafora). Studies have demonstrated a protective role for this compound in lowering blood glucose levels in alloxan-induced hyperglycaemic rats. A dose of 50–100 mg/kg of the body weight of the animals also resulted in a reduction in aglycated haemoglobin (HbA11C) levels 5 weeks after the study. The oxidative stress induced by high sugar levels and the animal body weight decreased [68]. Numerous studies have shown that mushroom-derived protein has a complete amino acid profile. As highlighted by Thatoi and Singdevsachan [69], its nutritional value is even greater than that of milk, meat, or egg proteins. The protein present in mushrooms can be characterised by a high content of essential amino acids and glutamic acid, aspartic acid, or arginine. Pop et al. [70] reported that the tramates versicolor contained as many as 18 types of amino acids like aspartic acid, threonine, serine, glutamic acid, glycine, alanine, valine, and leucine, which were identified. Furthermore, studies have confirmed the presence of ornithine, which is known for its particular physiological activity, and the non-protein neurotransmitter γ-aminobutyric acid (GABA) [71].

### 2.3. Lipids

The fat content in mushrooms varies depending on the species but can range from 0.1 to 5.9 g/100 g [19]. Among medicinal mushrooms, Cordyceps (*Ophiocordyceps sinensis* (Berk.) G.H. Sung, J.M. Sung, Hywel-Jones, and Spatafora) and Shiitake (*Lentinula edodes* (Berk.) Pegler) are the most abundant in fat. About 52–87% of the fat is made up of unsaturated fatty acids (UFAs) such as oleic (C18:1) and linoleic (C18:2) acids [1,25,71,72]. These acids predominate, for example, in Cordyceps (*Ophiocordyceps sinensis* (Berk.) G.H. Sung, J.M. Sung, Hywel-Jones and Spatafora). The minor fatty acid in this mushroom is saturated fatty acids, e.g., palmitic (C16:0) and stearic (C18:0) acids. Guo et al. [72] found that the fatty acid profile could vary depending on geographical origin in the example of cordyceps (*Ophiocordyceps sinensis* (Berk.) G.H. Sung, J.M. Sung, Hywel-Jones and Spatafora).

Comparative examinations between indoor-cultivated and wild *Ophiocordyceps sinensis* (Berk.) G.H. Sung, J.M. Sung, Hywel-Jones, and Spatafora, conducted by Guo et al. [72], demonstrated that the wild mushrooms were characterized by a higher PUFAs (Polyunsaturated Fatty Acids) content with indoor-cultivated mushrooms. Such fatty acids as oleic acid, hydroxydocosanoic acid, hydroxytricosanoic acid, hydroxytetracosanoic acid, and hydroxypentacosanoic acid are predominate in Shiitake (*Lentinula edodes* (Berk.) Pegler) mushrooms. Stearic acid, hydroxyhexacosanoic acid, linoleic acid, palmitic acid, hydroxyarachidic acid, hydroxyheneicosanoic acid, and hydroxy-tricosenoic acid are present in smaller amounts [17].

Linoleic acid is known to have anticancer effects on breast, colon, and prostate cancer; thus, as a natural source of this acid, medicinal mushrooms also exhibit such properties [73]. Furthermore, unsaturated fatty acids can be used for the production of tissue hormones and are useful in preventing excessive blood clotting.

### 2.4. Sterols

Mushrooms are also a source of sterols classified as bioactive compounds. The most common of these is ergosterol. This compound undergoes photolysis to vitamin D2 when exposed to UV radiation [74]. A study conducted by Zheng et al. [75] showed that ergosterol exhibited cytotoxicity towards acute promyelocytic leukaemia cancer cells and liver cancer cells. At the same time, the authors noted moderate antimicrobial activity against selected bacteria and fungi. A characteristic sterol named H1-A, which resembles testosterone and dehydroepiandrosterone in its structure, was isolated from Cordyceps. In vivo studies in mice have shown that this compound could be effective for the treatment of selected autoimmune diseases [76].

### 2.5. Polyphenols

Thanks to the presence of polyphenols, including mainly phenolic acids represented by benzoic acid and cinnamic acid derivatives, medicinal mushrooms can be attributed to antioxidant activity. As reported by Ahmed et al. [77], gallic, caffeic, and p-coumaric acids are the predominant phenolic compounds in mushrooms. Phenolic compounds that are present in mushrooms exhibit strong antioxidant properties [78]. They inhibit free radicals and limit peroxide decomposition, scavenge reactive oxygen species, and block the action of metals when catalysing oxidation reactions [79,80,81]. Thus, they prevent mutations of cellular DNA and reduce the processes of carcinogenesis [82]. Peng and Shahidi [17] analysed Chaga ethanol extracts and detected 111 different phenolic compounds, including phenolic acids, flavonoids, coumarins, quinones, and styrylpyrones. Flavonoids in medicinal mushrooms are represented by myricetin, rutin, naringenin, quercetin, morin, and hesperetin [83]. Research conducted by Sharpe et al. [78] showed that, among many medicinal mushrooms, Chaga (*Inonotus obliquus* (Ach. Ex Pers.) Pilát) had the highest polyphenolic content and the highest antioxidant activity. The total phenolic content in this mushroom was at 97 µmol GAE/mg, while the content in reishi (*Ganoderma lucidum* (Curtis) P. Karst.), shiitake, and turkey tail (*Trametes versicolor* (L.) Lloyd) was 21, 13, and 0.1 µmol GAE/mg, respectively. The water-ethanol extract from Chaga (*Inonotus obliquus* (Ach. Ex Pers.) Pilát) exhibited approximately five times higher antioxidant activity against DPPH than other mushrooms. Mushroom polyphenols exhibit multidirectional beneficial effects on the human body: anticancer, antioxidant, hypoglycemic, slowing down the aging process, and preventing the degenerative diseases of the nervous system and cardiovascular diseases. When used as a food additive, they reduce fat oxidation processes and extend the shelf life of products [79].

### 2.6. Terpenes and Terpenoids

Another group of compounds includes terpenes, with the general formula (C5H8)n, and terpenoids containing additional functional groups (-OH, -CHO, =CO, -COOH, -O-O-). Triterpenes are the main biologically active metabolites of terpenoid nature and are synthesized by *Ganoderma lucidum* (Curtis) P. Karst. and *Inonotus obliquus* (Ach. ex Pers.) Pilát. Data in the literature have reported that large amounts of these compounds, e.g., in reishi (*Ganoderma lucidum* (Curtis) P. Karst.) and chaga (*Inonotus obliquus* (Ach. ex Pers.) Pilát). Terpenes exert primarily anti-inflammatory effects. Triterpenes isolated from *Ganoderma lucidum* (Curtis) P. Karst. and *Inonotus obliquus* (Ach. ex Pers.) Pilát reduced the secretion of pro-inflammatory cytokines in macrophages (such as TNF-α, IL-1β, and IL-6) and the inflammatory mediators of nitric oxide (NO) and prostaglandin E2 (PGE2) [84,85]. Similarly, anti-inflammatory properties were exhibited by lanostane-type triterpene acids present in *Ganoderma lucidum* (Curtis) P. Karst., which, as shown by Akihisa et al. [85], inhibited the inflammatory process induced in mouse macrophages. In addition to anticholinesterase activity, the beneficial effects of mushroom terpenes have been reported in anticancer, antiviral, antimalarial, and antimalarial treatments [86,87]. The pharmacological effect of triterpenoids has been employed in the treatment of neurodegenerative diseases, including Alzheimer’s disease [88].

### 2.7. Vitamins and Minerals

The nutritional value of medicinal mushrooms is also related to their high vitamin and micronutrient content. The vitamins present in mushrooms are mainly fat-soluble vitamins, including A and E. as well as vitamin D2 (ergocalciferol) and provitamin D2 (ergosterol). Interestingly, medicinal mushrooms are considered to be the only non-animal raw material that contains vitamin D [27,89]. Thanks to their tocopherol content, medicinal mushrooms exhibit antioxidant properties [89]. In addition, medicinal mushrooms are a very good source of water-soluble B vitamins (B1, B2, B3, B6, B9, B12) and vitamin C [19]. The vitamin B12 found in medicinal mushrooms was an analogue of that found in beef, fish, and liver, indicating its highly bioavailable. Therefore, mushrooms can be a valuable addition to vegetarian and vegan diets [90,91]. Shitake is rich in vitamins that exhibit antioxidant properties such as A, E, and C [27]. Medicinal mushrooms are rich in valuable mineral elements, including K, P, Na, Ca, and Mg, and, in smaller amounts, Cu, Zn, Fe, Mo, and Cd [27,79]. Given the ability of fungi to accumulate such heavy metals as Cd, Pb, Ar, Cu, Ni, Ag, Cr, and Hg, it is important that they grow in the least contaminated environment possible [92].

## 3. Possibilities of Using Medicinal Mushrooms for Functional Food Production

Medicinal mushrooms and mushroom-derived preparations containing bioactive compounds are classified as nutraceuticals. According to the European Food Safety Agency, they can be used as supplements due to their health-promoting and disease-preventing activity [93]. The production of nutraceuticals requires a great deal of knowledge of the functional properties of individual mushroom species. Due to the possible presence of substances that are harmful to health, it is necessary to control the origin, cultivation conditions, and raw material processing in order to ensure the health and safety of nutraceutical products on the one hand and an adequate level of biologically active compounds on the other to guarantee the beneficial effects of its preparation on health [94]. Clinical studies have shown that the recommended dose of nutraceutical preparations varies depending on the diagnosis and the patient [95]. Currently, a variety of fungal preparations are commercially available, most commonly in the dry extract form. There is growing interest in exploring the possibility of using various medicinal mushroom preparations to develop functional foods. An example of a popular product with medicinal mushrooms such as Chaga (*Inonotus obliquus* (Ach. ex Pers.) Pilát), cordyceps (*Ophiocordyceps sinensis* (Berk.) G.H. Sung, J.M. Sung, Hywel-Jones & Spatafora), shiitake, lion’s mane (*Hericium erinaceus* (Bull.) Pers.) or reishi (*Ganoderma lucidum* (Curtis) P. Karst.) is coffee. Its consumption regulates blood pressure, prevents heartburn, stimulates mental performance, boosts energy, and strengthens the immune system and performance of the organism [96]. Some medicinal mushrooms have also been used to enrich cereals, meat, fish, and beverage products (Table 4). Of the mushrooms discussed so far, reishi (*Ganoderma lucidum* (Curtis) P. Karst.) and shiitake (*Lentinula edodes* (Berk.) Pegler) have been used most commonly. In all food products, the addition of dried and powdered mushrooms resulted in an increase in protein and the total and insoluble dietary fibre and significantly increased the micronutrient content [66]. The introduction of mushroom powder at 5% in such bakery products as bread and biscuits did not have adverse effects of their quality [97]. In the case of additions above 5%, a deterioration in texture was often noted not only in bread but also in pasta, yoghurt, and cured meats (Table 4). There is no information in the literature on the possibility of using turkey tail (*Trametes versicolor* (L.) Lloyd), cordyceps (*Ophiocordyceps sinensis* (Berk.) G.H. Sung, J.M. Sung, Hywel-Jones and Spatafora), lion’s mane (*Hericium erinaceus* (Bull.) Pers.), or Chaga (*Inonotus obliquus* (Ach. ex Pers.) Pilát) preparations for food enrichment. Given the high health-promoting potential of these mushrooms, further research into the possibility of developing new functional foods with the above-mentioned mushrooms is advisable. The effect on the addition of selected medicinal mushrooms on quality parameters and the chemical composition of food products is presented in Table 4. An important issue in the design and implementation of new food products is sensory quality. Scientific studies have shown that the addition of medicinal mushrooms to foods, especially in a crushed or powdered form, can have a negative effect on the taste, texture, flavour, colour, and appearance of products. The addition of alcoholic or aqueous mushroom extracts has a less negative impact on the sensory quality and, with a small amount (up to 4%), can even improve the selected sensory characteristics of products. 

## 4. Conclusions or Concluding Remarks

To date, a great deal of research has already been conducted into medicinal mushrooms; however, given the diversity of species and the amount of bioactive substances contained therein, this area still appears to be incompletely explored. It seems advisable to conduct research not only to isolate and identify the bioactive substances present in mushrooms but also to conduct clinical experiments to confirm the therapeutic effect of these substances. Such studies could facilitate a determination of the dose and duration of use for mushroom nutraceuticals. Toxicological studies confirming the safety of medicinal mushrooms are also needed. In the context of using medicinal mushrooms for the development of functional foods, it is important to study the interactions between the biologically active compounds present in mushrooms and food ingredients. It is important to bear in mind that the components present in the food matrix may act synergistically or antagonistically with mycochemicals, increasing or reducing their beneficial physiological effects, respectively. Based on the analysis of available information and scientific research, it can be concluded that the addition of medicinal mushrooms to foods, especially cereal products, can make their chemical composition more attractive due to their great health-promoting properties and the presence of biologically active compounds. Medicinal mushrooms are known for their potential to improve immunity, regulate metabolism, and prevent many diseases. The abundance of polysaccharides, polyphenols, amino acids, and vitamins in medicinal mushrooms is a valuable source of biologically active compounds that can contribute to maintaining the health and well-being of the body. At the same time, further scientific research is needed to confirm these benefits and develop optimal methods for the addition of medicinal mushrooms to foods, taking into account technological, sensory, and food safety aspects.

## Figures and Tables

**Table 1:** The chemical composition of medicinal mushrooms (g/100 g dried mushrooms).

Common Name	Latin Name	Moisture	Protein	Carbohydrates	Lipids	Dietary Fibre	Ash	The Literature Source
Reishi	*Ganoderma lucidum* (Curtis) P. Karst.	7.5–12.99	13.3–23.6	42.8–82.3	3–5.8	14.81	4	[19,22,23]
Lion’s Mane	*Hericium erinaceus* (Bull.) Pers.	7.03 *	22.3	57.0	3.5	3.3–7.8	7.1	[19,23]
Chaga	*Inonotus obliquus* (Ach. ex Pers.) Pilát	3.5	2.4	10.3	1.7	67.5	n.d.	[20]
Cordyceps	*Ophiocordyceps sinensis* (Berk.) G.H. Sung, J.M. Sung, Hywel-Jones and Spatafora*prior name Cordyceps sinensis*	3.5 *	21.9–23.1	24.2–49.3	5.5–8.2	7.7	13.13	[19,23]
Shiitake	*Lentinula edodes* (Berk.) Pegler	7.14	17.2–27.09	38.1–66.0	1.26–2.95	46.19–49.09 (IDF: 40.7–44.2 and SDF: 1.95–8.4)	6.05–6.73	[24,25,26,27]
Turkey Tail It also known as:, Cloud mushroom, Yun Zhi, Kawaritake	*Trametes versicolor* (L.) Lloyd	-	11.07	-	1.35	-	-	[28]

(-)—no data; (*)—unpublished own research, IDF—water-insoluble dietary fibre, SDF—water-soluble dietary fibre.

**Table 2:** Beta-glucan content of different medicinal mushrooms [31,44,48].

Common Name	Latin Name	Content of β-Glucans (g/100 g d.b.)
Reishi	*Ganoderma lucidum* (Curtis) P. Karst.	4.3–23.6
Lion’s Mane	*Hericium erinaceus* (Bull.) Pers.	35.3
Chaga	*Inonotus obliquus* (Ach. ex Pers.) Pilát	8.5
Shiitake cap/steam	*Lentinula edodes* (Berk.) Pegler	20.0/25.3
Turkey tail	*Trametes versicolor* (L.) Lloyd	60.79
Cordyceps	*Ophiocordyceps sinensis* (Berk.) G.H. Sung, J.M. Sung, Hywel-Jones and Spatafora *prior name Cordyceps sinensis*	3.79

**Table 3:** Bioactive components in medicinal mushrooms and their health-promoting effects.

Common Name	Latin Name	Compounds with Bioactive Potential	Health-Promoting Effects	References
Reishi	*Ganoderma lucidum* (Curtis) P. Karst.	Polysaccharides Glycoproteins (lectins) Phenols Steroids Triterpenoids Nucleotides Fatty acids Vitamins Minerals	Anti-inflammatory Anticancer Antiviral (including HIV) Antimicrobial Hypotensive effect Cardiotonic Immunomodelling Nephrotonic Hepatoprotective Neurotonic Anti-asthmatic	[21] ^a^, [51], [52] ^a,b^, [53]
Lion’s Mane	*Hericium erinaceus* (Bull.) Pers.	Hericerins, Erinacins, Glycoprotein, Polysaccharides Beta-glucans, Sterols, Lactone, Fatty acids Volatile compounds (e.g., hexadecanoic acid, linoleic acid, phenylacetaldehyde, benzaldehyde)	Anticancer, Antioxidant, Anti-ageing, Imunomodelling, Neurotonic, Anti-asmatic, Hypoglycemic effects Hypocholesterolemic effects	[46] ^a,b^, [53], [54] ^a,b^
Chaga	*Inonotus obliquus* (Ach. ex Pers.) Pilát	Polysaccharides Fatty acids Hydroxy acids Poliphenols (phenolic acids, flavonoids, coumarins, quinones, and styrylpyrones) Triterpenoids (lanosterol) Steroids (ergosterol and ergosterol peroxide)	Antioxidant, Anti-ageing, Antimicrobial activity, Antitumor activity, Anti-inflammatory hypoglycemic effect, Antilipidemic effect, Antiglication effect, Immunoregulatory Cardioprotective effects	[14] ^b^, [17,54,55]
Cordyceps	*Ophiocordyceps sinensis* (Berk.) G.H. Sung, J.M. Sung, Hywel-Jones & Spatafora *prior name Cordyceps sinensis*	Cordycepin (purine alkaloid) Cordymin (peptide) Adenosine Cordycepic acid (d-mannitol) Trehalose Polysaccharide Beta-glucans Saponins Polyunsaturated fatty acids, Ergosterol δ-tocopherol Hydroxybenzoic acid	Antitumor, Hypoglycemic effect Hypocholesterolemic effect, Anti-inflammatory, Antioxidant, Antiaging activity, Antimicrobial activity, Anticonvulsant activity, Cardiovascular protection (reduces cardiac arrhythmia and chronic heart failure)	[49], [56] ^ab^, [57] ^a^, [58] ^a^, [59] ^a^
Shiitake	*Lentinula edodes* (Berk.) Pegler	Polysaccharides, Beta-glucans (lentinan) Glycoproteins, Phenols, Steroids, Terpenoids, Nucleotides	Immune-enhancing effects, Antitumor, Antioxidant, Antiaging activity, Antimicrobial activity, Hypocholesterolemic effect, Reduction in blood pressure	[26], [27] ^a^, [60]
Turkey Tail It also known as: Cloud mushroom, Yun Zhi, Kawaritake	*Trametes versicolor* (L.) Lloyd	Polysaccharopeptide (PSP) and polysaccharide K (PSK) (1,3)(1,6)-β-d-glucans, Poliphenols (phenolic acids: p-hydroxy benzoic, protocatechuic, vanillic, and homogentisic), Vitamin B, Fatty acids (linoleic, oleic, stearic, linolenic)	Antitumor Immunoregulatory, Antioxidant activity Prevent obesity, Antimicrobial, Antidiabetic AChE inhibitorY	[28,61], [62] ^b^

^a^—in vivo studies. ^b^—in vitro studies.

**Table 4:** Use of medicinal mushrooms for food enrichment.

Common Name	Latin Name	Product/ Size of Additive	Impact on Chemical Composition (~) Lack of Impact (↓) Decrease (↑) Increase	Impact on Quality Parameters	References
Reishi	*Ganoderma lucidum* (Curtis) P. Karst.	Smoked fish sausage 1% of crushed mushroom	(↑) Antioxidant properties (↑) Total phenol content: + (↓)Moisture: − (↑) Ash: + (↑) Protein: + (↓)Fat: − Fiber: +	(↑) Shelf life (↓) Texture Sensory evaluation: (↓) flavour, (↓) colour, (↓) taste, (↓) texture, (↓) appearance, (↓) overall	[98]
		1% of water extract	(↑) Antioxidant properties (↑) Total phenol content (↓) Moisture: − (↑) Ash (↓) Protein: − (~) Fat (↑) Fiber: +	(↑) Shelf life (↑) Texture - Sensory evaluation: (↑) flavour, (↑) colour, (↑) taste, (↑) texture, (~) appearance, (↑) overall	
		0.25% of spore	(↑) Antioxidant properties (↑) Total phenol content (↓) Moisture: − (↑) Ash (↑) Protein (↑) Fat (↓) Fiber	(↑) Shelf life (~) Texture Sensory evaluation: (↓) flavour, (↓) colour, (↓) taste, (↓) texture, (~) appearance, (↓) overall	
Reishi	*Ganoderma lucidum* (Curtis) P. Karst.	Pilzner beer 0.1–1.5 mL/L of alcohol extract		Sensory evaluation: (~) aroma (↑) taste (↑) body (↑) bitterness (↑) liveliness (↑) overall impression	[99]
Reishi	*Ganoderma lucidum* (Curtis) P. Karst.	Emulsion Type Sausage 1% of dried fruiting bodies	(↑) Antioxidant properties	Sensory evaluation: (~) texture − (↓) taste (↓) Colour − (↓) Smell (↓) Acceptability (~) Peroxide value (↑) Microbiological analysis +	[100]
Reishi	*Ganoderma lucidum* (Curtis) P. Karst.	Bread 2/4/6/8% water extract		(↑) Baking loss (↓) Bitterness Sensory evaluation: (↑) 2–4%, (↓) 6–8% Texture: (~) 2–4% (↓) 6–8% −	[101]
Reishi	*Ganoderma lucidum* (Curtis) P. Karst.	Yoghurt 2% Industrial waste (residues from aqueous extraction)	(↑) anti-coli effect, (↑) against *E. coli*	(↓) Texture (↓) Taste	[102]
Reishi	*Ganoderma lucidum* (Curtis) P. Karst.	Semolina pasta enriched with 2.5 and 5% of mushroom powder	(~) Phenolic compounds (↑) ABTS antiradical properties (↑) Syringic acid (~) β-glucan content (~) Anticancer properties	Not analyzed	[103]
Lion’s Mane	*Hericium erinaceus* (Bull.) Pers.	Semolina pasta enriched with 2.5 and 5% of mushroom powder	(~) Phenolic compounds (~) Antioxidant properties (~) ABTS antiradical properties (↑) Vanilin (~) β-glucan content (~) Anticancer properties	Not analyzed	[103]
Shiitake	*Lentinula edodes* (Berk.) Pegler	Biscuits with mushroom powder 10%	(↑) Protein (↑) Mineral (Fe, P, Zn, Ca) (↑) Total and insoluble dietary fibre	Sensory evaluation: (~) aroma, (~) colour, (~) texture, (~) shelf life	[104]
Shiitake	*Lentinula edodes* (Berk.) Pegler	Bread enriched with 5–15% addition of mushroom powder	(↑) Dietary fiber	Bread dough: (↑) water absorption; (↓) development time; (↓) stability; >5% decreased the dough strength Bread quality physical: (↓) loaf height; (↑) moisture content; (↓) specific volume; >5% (↑) bread’s gumminess; >5% bread’s (↑) hardness; (↓) porosity	[97]
		Pork patties 0–6% addition to mushroom powder	Not analyzed	(↑) texture +; (↑) juiciness +; (↑) moisture +	[105]
		Semolina pasta enriched with 5–15% addition of mushroom powder	Not analyzed	(↑) Cooking loss (~) Water absorption; (~) Moisture content; (~) Tensile strength; (↑) Firmness	[106]

## Data Availability

Data sharing not applicable.
